# *Hydrangea serrata* Hot Water Extract and Its Major Ingredient Hydrangenol Improve Skin Moisturization and Wrinkle Conditions via AP-1 and Akt/PI3K Pathway Upregulation

**DOI:** 10.3390/nu15112436

**Published:** 2023-05-24

**Authors:** Ji Hye Yoon, Sang Hee Park, Si Eun Yoon, Seong Yoon Hong, Jun Bae Lee, Jongsung Lee, Jae Youl Cho

**Affiliations:** 1 Department of Biocosmetics, Sungkyunkwan University, Suwon 16419, Republic of Korea; 2 Innovation Lab., Cosmax R&I Center, Seongnam 13486, Republic of Korea; 3 Department of Integrative Biotechnology, Biomedical Institute for Convergence at SKKU (BICS), Sungkyunkwan University, Suwon 16419, Republic of Korea

**Keywords:** *Hydrangea serrata*, hydrangenol, clinical trial, wrinkles, moisturizing, AP-1

## Abstract

*Hydrangea serrata* is a plant grown in Korea and Japan with a particular natural compound, hydrangenol. *H. serrata* has been researched for its anti-fungal properties, and ability to attenuate allergies and promote muscle growth. Its ability to reduce skin dryness is poorly understood. For that reason, we investigated whether *H. serrata* hot water extracts (Hs-WE) can moisturize keratinocytes. In clinical studies (Approval Code: GIRB-21929-NY and approval Date: 5 October 2021), skin wrinkles and skin moisturizing levels were improved in subjects applying 0.5% Hs-WE compared to the placebo group. We confirmed the components of Hs-WE from the LC/MS-MS analysis. Hs-WE and hydrangenol did not show cytotoxicity in HaCaT cells at all concentrations. Cell growth was also promoted by Hs-WE (5–20 µg/mL) and hydrangenol (15–60 µM) in a wound healing assay. Skin moisturizing factors were upregulated by the presence of Hs-WE or hydrangenol, and the hyaluronidases (HYAL) were inhibited at the mRNA level. Meanwhile, COL1A1 was increased by the presence of Hs-WE or hydrangenol. MAPK, AP-1, and Akt/PI3k signaling proteins, which are associated with cell proliferation and moisturizing factors, were increased by the administration of Hs-WE and hydrangenol. Has-1, 2, and 3 levels were enhanced via JNK when using the inhibitors of MAPK proteins and Hs-WE and hydrangenol, respectively. Taken together, Hs-WE could be used as cosmeceutical materials for improving skin conditions.

## 1. Introduction

Aging is a high-profile issue due to increasing skin water loss, wrinkles, dryness, and dermal atrophy [1,2,3]. Aging can be categorized based on two main causes: extrinsic and intrinsic aging [4]. While extrinsic aging is accelerated by external environmental factors such as air pollution, reactive oxidative species (ROS), fine dust, and UV exposure [5,6], intrinsic aging progresses are due to physiological and genetic factors. Skin will always be exposed to external conditions in life, and humans cannot prevent physiological aging over time with current technologies. Therefore, aging is considered an inevitable natural phenomenon. However, it would be desirable to arrest accelerated aging [7]. There are numerous research efforts focused on anti-aging or reversing aging. Most of these studies have focused on improving the symptoms of aging such as skin hydration, and decreasing collagen and skin junctions. Skin hydration is one of the major indicators of intrinsic aging. It is related to intracellular moisture levels in the skin, and skin dryness is the main cause of skin wrinkles. Methods for retaining skin moisture include upregulating skin moisturizing factors like hyaluronic acid synthase 1, 2, and 3 (Has-1, 2, and 3) and the factors associated with skin tight junctions, such as involucrin (INVN), occludin (OCLN), transglutaminase1 (TGM1), and filaggrin (FLG), or downregulating hyaluronidase (HYAL). These factors can be increased via the activator protein1 (AP-1) pathway [8,9].

AP-1 is the transcription factor composed of c-Fos and c-Jun, and these subunits bind to AP-1 binding sites [10,11]. This dimeric complex synthesizes the genes involved in cell proliferation, differentiation, and survival through the transcription process [12]. Moreover, these proteins are activated by the upper signaling factor, mitogen-activated protein kinase (MAPK) pathway proteins. p38, extracellular signal-regulated kinases (ERK), and c-Jun NH2-terminal kinases (JNK) have been identified in the eukaryotic MAPK pathway. Cell differentiation and proliferation like the AP-1 pathway play an important role in cell transduction [13,14,15,16,17]. According to the results of a previous study, plant extracts containing phytochemicals can exert a skin moisturizing effect through the AP-1 protein pathway [18,19].

Dermal change is also one of the representative aging symptoms. The remarkable phenomena such as the degradation of collagen and elastin fibers or low synthesis of procollagen co-occur in the dermis. For that reason, fine wrinkles are formed in the skin over time [20,21,22]. The collagen fibers could be degraded by matrix metalloproteinases (MMPs) which can proceed in aged skin [23,24,25,26]. Furthermore, senescent fibroblasts cannot renew the new collagen fibers as collagen synthesis is degraded in aged fibroblasts [27,28]. For that reason, we investigated the effects of plant extracts on the skin, focusing specifically on whether they help in the synthesis of pro-collagen and moisturize the skin.

*Hydrangea serrata* (Acumina) is a plant grown in Korea and consumed as a tea in Korea and Japan [29]. The extract of this plant has been known to display anti-obesity and anti-photoaging activities [30,31]. It has been reported that *H. serrata* has phytochemicals including hydrangenol, macrophylloside, phyllodulcin, and phyllodulcin [32,33]. Of these, hydrangenol was found to display anti-allergic, anti-fungal, anti-inflammatory, anti-diabetic, and anti-angiogenic effects as one of the natural dihydroisocoumarins [34,35,36,37,38]. Moreover, the effects of *Hydrangea serrata* hot water extract have also been researched including their anti-inflammation activity, anti-aging effect, anti-obesity activity, and ability to aid in muscle synthesis [39,40,41,42,43]. However, there is no study about intrinsic aging; through our study, we investigated the skin moisturizing and anti-wrinkle potential of *Hydrangea serrata* extracts.

## 2. Materials and Methods

### 2.1. Preparation of Extract, Chemicals, and Antibodies

Leaves of *H. serrata* (1 kg) were extracted for 5 h from 15 L of distilled water at 98 °C. The extracts were filtered through a 10 µm filter and were concentrated using a decompression concentrator at 180–200 °C to produce dried powder concentrates. The powder was dissolved in DMSO at a concentration of 100 mg/mL. A Human keratinocyte cell line (HaCaT cells) was purchased from the American Type Culture Collection (ATCC, Rockville, MD, USA). Dulbecco’s modified eagle’s medium (DMEM), and penicillin/streptomycin solution (PC/SM) were obtained from Hyclone (Logan, UT, USA). Phosphate-buffered saline (PBS) was purchased from Samchun Pure Chemical Company (Seoul, Republic of Korea). Trypsin-EDTA (0.25%), lipofectamine 2000, minimal essential medium (opti-mem), and fetal bovine serum (FBS) were purchased from Gibco (Grand Island, NY, USA). Sigma Aldrich (St. Louis, MO, USA) was the source for dimethyl sulfoxide, D-luciferin, β-galactosidase, isopropanol, and 1-bromo-3-chloropropane. Trizol reagent^®^ was purchased from Molecular Research Center, Inc. (Cincinnati, MA, USA). Macrogen (Seoul, Republic of Korea) synthesized the primer used in the qRT-PCR assay. The primary antibodies, including JNK, ERK, p-ERK, p38, p-p38, c-Jun, p-c-Jun, p-c-Fos, c-Fos, PI3K, p-PI3K, PDK-1, p-PDK-1, AKT, p-AKT HAS-1,2,3, p-JNK, b-actin, and COL1A1, were purchased from Santa Cruz Biotechnology (Dallas, TX, USA), Cell Signaling Technology (Beverly, MA, USA), or Novus Biological (Toronto, ON, Canada). The HRP-conjugated goat anti-rabbit antibody and horse anti-mouse antibody were obtained from Abcam (Cambridge, UK). Ab Frontier (Seoul, Republic of Korea) was the source for the enhanced chemiluminescence Western blotting substrate (ECL).

### 2.2. Cell Culture

The HaCaT cells and human dermal fibroblast (HDF) cells were cultured in DMEM media containing 10% fetal bovine serum and 1% antibiotics (PC/SM). The cells were incubated with 5% CO^2^ at 37 °C. At 70% confluency, the cells were harvested using a trypsin–EDTA solution and were cultured at an appropriate density on plates.

### 2.3. Cell Viability

HaCaT cells were plated at a density of 5 × 10^4^ cells in each well of 96-well plates filled with 100 µL of media and were incubated at 5% CO^2^ and 37 °C for 24 h. The cells were treated with Hs-WE (0 to 200 µg/mL) or hydrangenol (0 to 40 µg/mL) for 24 h. After 24 h of incubation, the cells were treated with 10 µL of 3-(4,5-dimethylthiazol-2-yl)-2,5-diphenyltetrazolium bromide (MTT) solution (5 mg/mL in distilled water). MTT stopping solution (0.1N HCl in 10% SDS solution) was added to a tenfold volume of MTT solution to stop the reaction of the mitochondria. The absorbance was detected through a spectrometer at 570 nm.

### 2.4. Wound Healing Assay

The HaCaT cells were plated in 48-well plates at a density of 5 × 10^4^ cells in 500 µL of media per well and were incubated for 24 h. The cells were scratched by a plastic scratcher and treated with WHS extract (0 to 20 µg/mL) or hydrangenol (0 to 60 µg/mL). Pictures were taken with the microscope after incubating with the extract for 0, 24, and 48 h.

### 2.5. qRT-PCR

The qRT-PCR assay was conducted to estimate the mRNA levels of moisturizing factors, including *HAS-1*, *HAS-2*, and *HAS-3*; skin barrier factors, such as *INVN*, *TGM*-1, *OCLN*, and *FLG*; hyaluronidases, such as *HYAL*-*1*, *HYAL-2*, *HYAL-3*, and *HYAL-4*; keratins, such as *Ker5*, *Ker6*, and *Ker16*; and pro-collagen Type 1, such as *COL1A1*. The HaCaT cells and HDF cells were plated and treated as described above. To isolate the mRNA, the TRIzol reagent was added to each cell. The qRT-PCR experiment was performed using our previous study’s protocols [18]. The primers used in this experiment are listed in Table 1.

### 2.6. Luciferase Reporter Assay

The HaCaT cells were seeded in 24-well plates with 1 mL of cells in DMEM without antibiotics. The cells were transfected with lipofectamine 2000 and 1 µg/mL of AP-1-Luc plasmid with β-galactosidase in OPTI-MEM media. Next, the cells were treated with the extract from 0 to 20 µg/mL and the compound from 0 to 60 µg/mL. A volume of 300 µL of lysis buffer was added to each well for cell lysis. After freezing at −70 degrees for 3 h, the cell lysis and β-galactosidase complex, and d-luciferin complex were detected through a spectrometer at 405 nm or via luminescence.

### 2.7. Western Blotting

The HaCaT cells and HDF cells were plated at the same density as described above, and the cell lysates were transferred to a new e-tube using PBS after treating with Hs-WE and hydrangenol for 24 h. Then, the cells were centrifuged at 3000 rpm for 3 min. The cell lysates were reacted with cell lysis buffer containing 2 mM EDTA, 150 mM NaCl, 20 mM Tris-HCl pH 7.5, 20 mM NaF, 2% NP-40, and 50 mM β-glycerol phosphate with 2 µg/mL of pepstatin, leupeptin, and aprotinin. The mixtures were then centrifuged at 12,000 rpm for 10 min after the reaction. The supernatants were used for Western blotting and separated by 12% sodium dodecyl sulfate-polyacrylamide gel electrophoresis (Bio-Rad, Hercules, CA, USA). The samples were transferred to a PVDF membrane (Merck Millipore) and blocked with a bovine serum albumin (BSA) 3% (*w*/*v*) solution. The membrane was incubated overnight with primary antibodies (1:2500 dilution) at 4 °C and was washed for 3 min three times with a TBST solution. Then, it was incubated for 2 h with secondary antibodies at room temperature. The protein bands were detected with enhanced chemiluminescence reagents.

### 2.8. Clinical Trial

This clinical study was conducted in a double-blind, randomized, and placebo-controlled design. The Global Medical Research Center (Seoul, Republic of Korea) conducted the clinical trial from 1 October 2021 to 16 November 2021 (NCT05872113). The subjects were recruited as healthy females aged 30–59 years (*n* = 22 people, 9 persons in their 40s and 12 persons in 50s with an average age of 49.238 years.) who fit the criteria. The inclusion and exclusion criteria are given in Table 2. The subjects who met the following criteria were excluded in the middle of the experiment: (1) the subject voluntarily presented her intention to discontinue participation in the study during the period, (2) observation of skin disease or adverse reaction, (3) excessive UV exposure of the test site, (4) results were judged to be impaired due to excessive drinking or smoking, and (5) subjects who had difficulty continuing the test and attending follow up appointments due to the subject’s circumstances. The cream was applied to the faces of subjects: the 0.5% Hs-WE cream was applied to the left facial area, while the placebo was applied on the right. The cream was made in two types (Table 3 and Table 4), one was 0.5% Hs-WE cream and the other was a placebo for estimating the effects of the Hs-WE. All subjects applied this cream two times per day for 4 weeks. The questionnaire for testing the effects was administered before using the cream, after 2 weeks, and after 4 weeks. Skin wrinkles were approximated by visual assessment using an Antera 3D CS (Miravex, Ireland) and skin moisturizing level was measured using a Corneometer CM825 (Courage and Khazaka, Köln, Germany). The study protocol was approved by the Institutional Review Board of the Global Medical Research Center (Approval Code: GIRB-21929-NY and approval date: 5 October 2021).

### 2.9. LC-MS/MS Analysis

Liquid chromatography-tandem mass spectrometry was carried out by COSMAX (Sungnam, Republic of Korea) to identify the components of the Hs-WE. This experiment was conducted with standard compounds such as gallic acid, kaempferol, vanillic acid, and resveratrol. The analysis used the instrument TSQ Altis™ Plus Triple Quadrupole Mass Spectrometer (Thermo Scientific, Waltham, MA, USA) with acetic acid (Merck, Rahway, NJ, USA) in MilliQ water and acetonitrile (J.T. Baker, Phillipsburg, NJ, USA) as the mobile phase. The amount and species of each compound was assessed based on the peak of the graph.

### 2.10. Statistical Analysis

All the data are shown as mean ± standard deviation (SD) for at least 3 replicates of independent trials. The Mann–Whitney U test was used to establish the statistical difference between individual experimental groups. In the clinical study, statistical analysis was verified using the IBM SPSS statistics 25.0 program. After the normality test, a one-way ANOVA test with paired samples t-test or Wilcoxon signed rank test was performed. Statistical values are written in terms of *p* < 0.05 (#, * *p* < 0.05; ##, ** *p* < 0.01).

## 3. Results

### 3.1. Skin Moisturizing and Anti-Wrinkle Effects of Hs-WE

**Figure 1:**
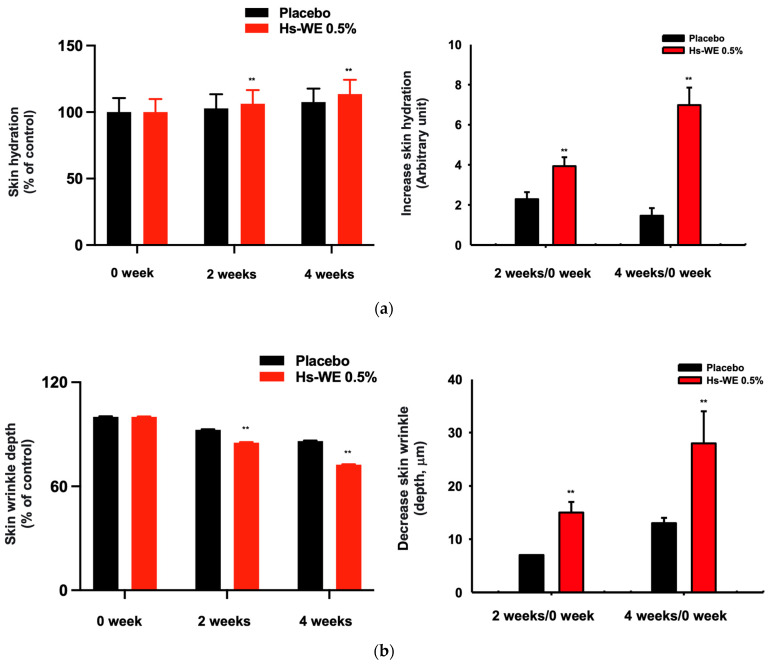

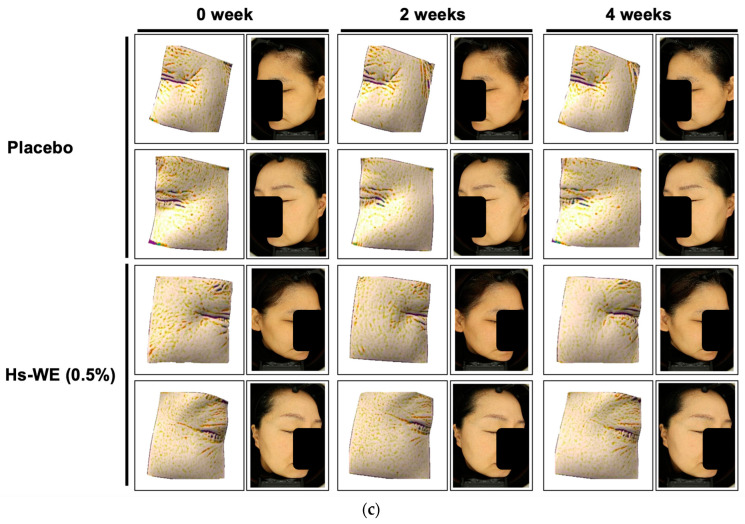
Anti-wrinkle effect of Hs-WE in clinical trial. (**a**,**b**) The skin hydration level and depth of wrinkles were detected through visual assessment using Antera 3D CS (Miravex, Ireland), and the value was calculated through comparison between application of Hs-WE and placebo. (**c**) The visualization of skin wrinkles from subjects who applied the sample to their faces. The pictures were taken at 0 week, 2 weeks, and 4 weeks. The measured values represent the mean ± standard deviation (SD) of three independent experiments. ** *p* < 0.01, compared with placebo-treated side of face using one-way ANOVA with paired samples t-test or Wilcoxon signed-rank test.

Twenty-two subjects were initially enrolled in the clinical trial. One subject was excluded by non-compliance with the plan of the trial. All subjects used the placebo sample on the left side of their face and the investigational sample on the right side of their face. The ingredients used to produce the samples for the clinical study are provided in Table 3 and Table 4. The moisturizing levels were measured before use (0 week), after 2 weeks, and after 4 weeks. The crow’s feet of the subjects were also measured at the same time points as the skin moisture level test. First, the improvements in wrinkles were more significant in the test sample than in the placebo sample (Figure 1a and Table 5). All products significantly enhanced the moisture levels of the skin. The skin moisturizing value increased when treating with the investigational sample compared to applying the placebo sample (Figure 1b and Table 6). As shown in Figure 1c, the skin wrinkles were attenuated by the test sample. Moreover, there were no reports of any adverse reactions during the use of the test products, so all the samples were deemed safe. Based on these results, Hs-WE could enhance skin moisture levels and also attenuate wrinkles in the skin.

### 3.2. Hs-WE Shows No Toxicity in HaCaT Cells

**Figure 2:**
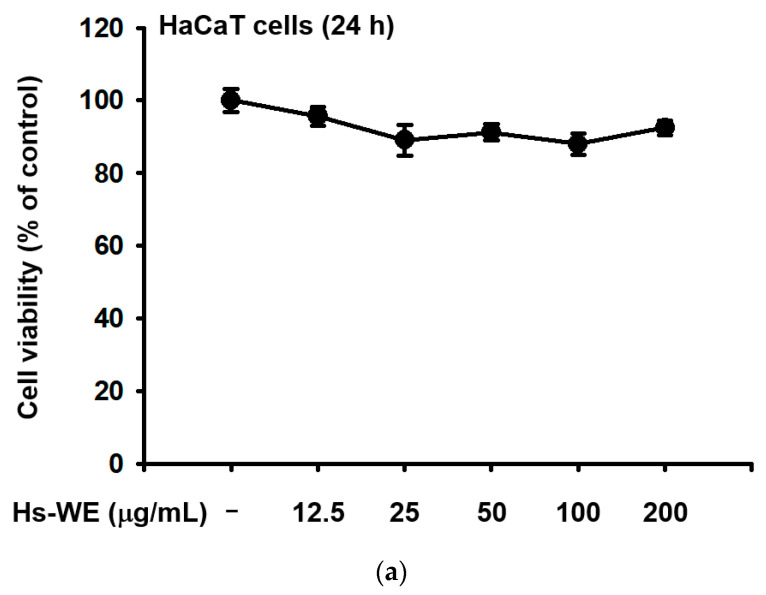

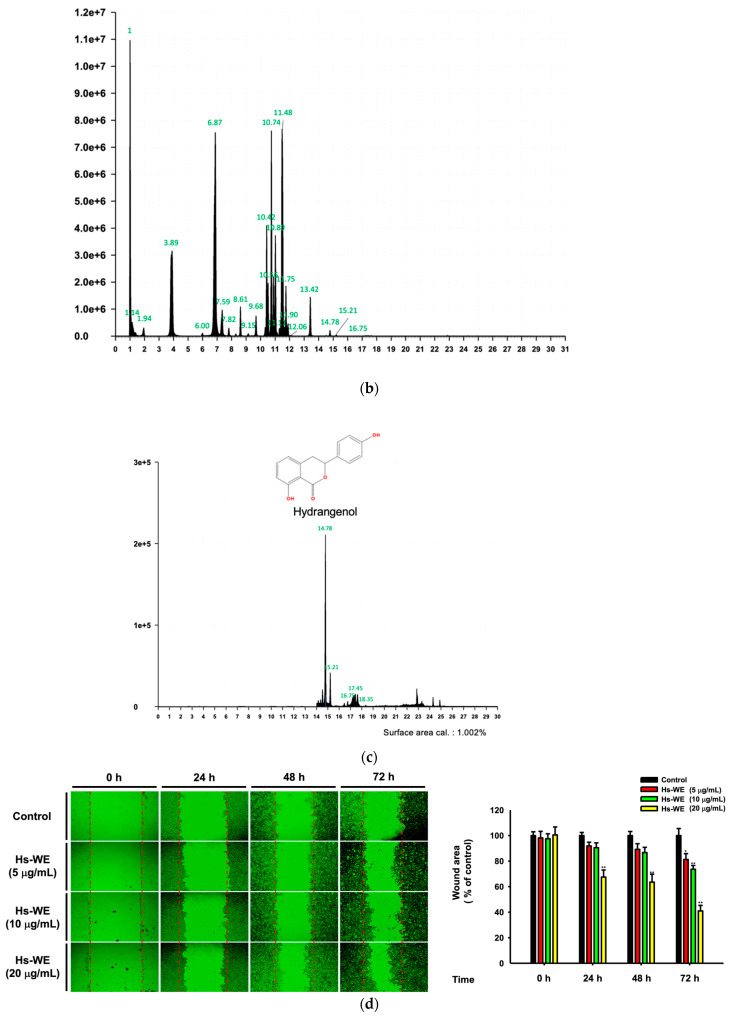
Effect of Hs-WE on the viability and wound healing of HaCaT cells and its LC/MS-MS profile. (**a**) Cell viability was detected at 570 nm using the MTT assay. The assay was performed on Hs-WE-treated HaCaT cells in 96-well plates. (**b**) The LC/MS-MS analysis of Hs-WE. (**c**) LC/MS-MS analysis to determine the hydrangenol content in Hs-WE. (**d**) The potential proliferation effects of Hs-WE were measured using a wound healing assay. The media including Hs-WE were changed after imaging. The area was detected using ImageJ software (Ver.: 1.53J, NIH, Bethesda, MD, USA). Values represent the mean ± standard deviation (SD) of three independent experiments. * *p* < 0.05, ** *p* < 0.01, compared with Hs-WE-treated group using the Mann–Whitney U test.

The MTT assay was conducted to verify the toxicity of the Hs-WE. As shown in Figure 2a, there was no toxicity in HaCaT cells at any tested concentrations. In the LC-MS quantitative analysis, there were many secondary metabolites such as chlorogenic acid, protocatechuic acid, rutin, hyperoside, GABA, resveratrol, loganin_A, isoquercitirin, gallic acid, p-coumaric acid, astraglin, caffeic acid, ferulic acid, salicylic acid, vanillic acid, kaempferol, and hydrangenol (Figure 2b and Table 7). In this extract, 1.002% hydrangenol was detected, which is considered a standard compound of hydrangeaceae (Figure 2c). A wound healing migration assay was performed to determine whether the Hs-WE affects cell proliferation. Healing activity was observed for all the Hs-WE-treated conditions after 72 h of incubation. Moreover, high levels of cell proliferation were observed at high concentrations, and cell proliferation was significantly increased in a dose-dependent manner compared to the control group (Figure 2d). In summary, Hs-WE could aid cell growth with no cytotoxicity. 

### 3.3. Hs-WE Skin Moisturizing Potential at the Transcription Level

**Figure 3:**
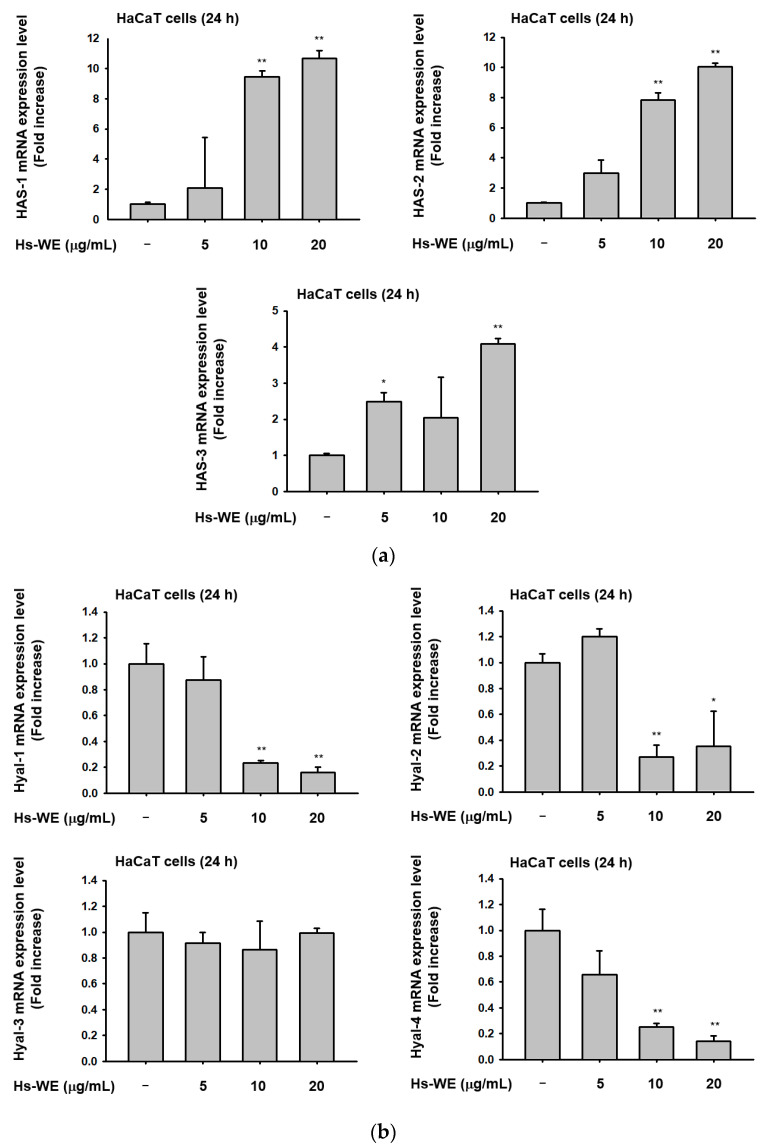

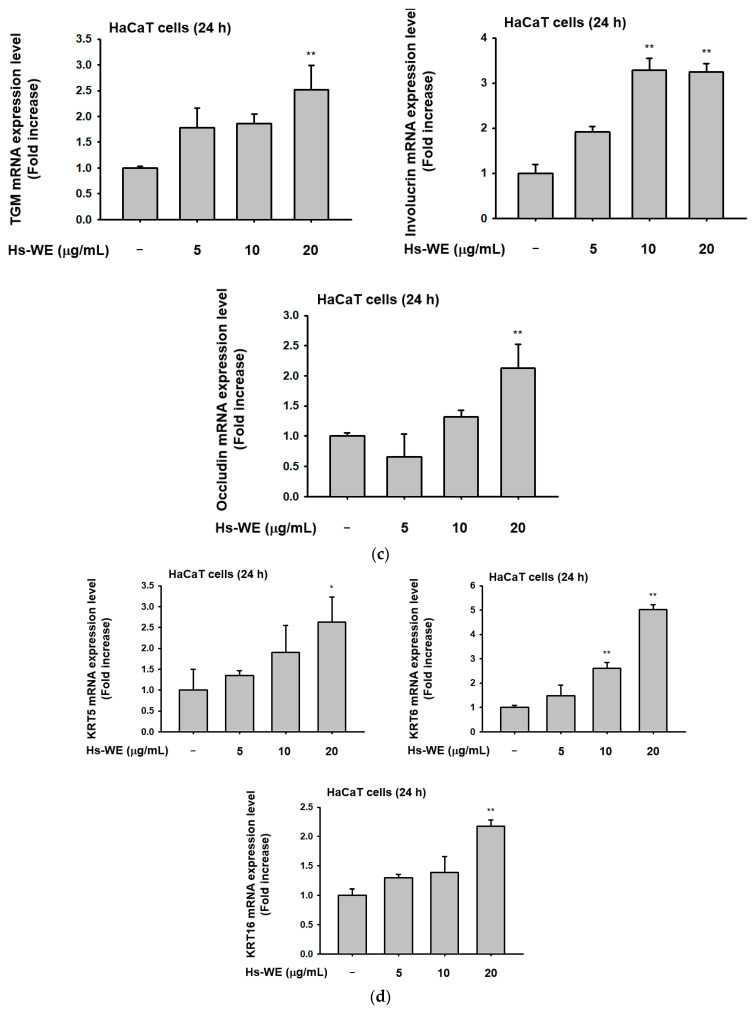

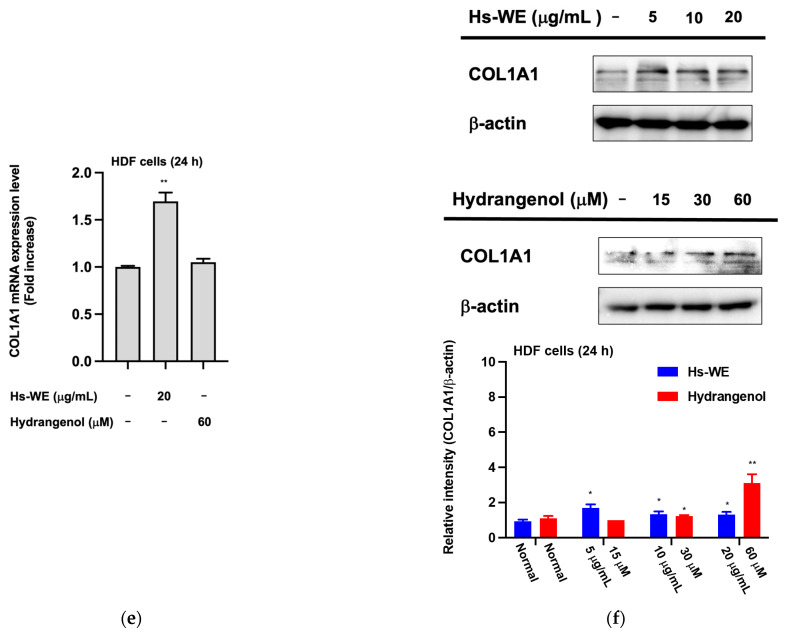
Effect of Hs-WE on the expression of moisturizing factors, tight junction factors, and collagen in skin. (**a**–**d**) HaCaT cells were treated with Hs-WE for 24 h. After isolating the mRNA, the synthesized cDNA was used in a PCR assay. Shown are the expression levels of genes associated with hyaluronic acid synthase (**a**), hyaluronidase (**b**), skin junctions (**c**), and epidermal proliferation (**d**). (**e**) The COL1A1 expression of HDF cells in the presence of Hs-WE (20 µg/mL) or hydrangenol (60 µM) was examined by qRT-PCR analysis. (**f**) Protein level of COL1A1 in HDF cells after treatment with Hs-WE (5–20 µg/mL) or hydrangenol (15–60 µM) was detected by Western blotting analysis. Values represent the mean ± standard deviation (SD) of three independent experiments. * *p* < 0.05, ** *p* < 0.01 compared with Hs-WE-treated group using Mann–Whitney U test.

We performed a qRT-PCR assay from Hs-WE-treated HaCaT cells to determine the skin moisturizing mechanism of the Hs-WE. The action of the Hs-WE on skin keratinocytes was confirmed through the qRT-PCR assay. Hyaluronic acid synthase as well as components of the skin barrier were upregulated under Hs-WE treatment (Figure 3a). In contrast, the mRNA levels of hyaluronidases such as *HYAL*-*1*, *HYAL-2*, and *HYAL-3* were downregulated in the Hs-WE-treated group in a dose-dependent manner (Figure 3b). Additionally, *TGM*, *OCLN*, INVN, and FLG were significantly upregulated to high concentrations in HaCaT cells when treated with the Hs-WE (Figure 3c). The wound-healing effects of the Hs-WE could be supported by confirming that the transcription levels of *Ker5*, *Ker6*, and *Ker16* were improved with Hs-WE treatment (Figure 3d). Moreover, we examined the level of collagen type I in HDF cells after treatment with Hs-WE to support the results of the clinical study. The mRNA expression of *COL1A1* was increased at higher concentrations of Hs-WE (20 μg/mL) (Figure 3e). Meanwhile, the protein level of *COL1A1* was revealed to be upregulated by the presence of Hs-WE or hydrangenol (Figure 3f). Overall, it was found that Hs-WE could improve skin hydration by upregulating the mRNA levels of the genes involved in keratin synthesis and hyaluronic acid synthesis, and downregulating the hyaluronidases. Furthermore, it was revealed that the Hs-WE could induce fibroblasts to synthesize procollagen-1 through transcriptional activation.

### 3.4. Hs-WE Upregulates the Expression of AP-1 Pathway Proteins

**Figure 4:**
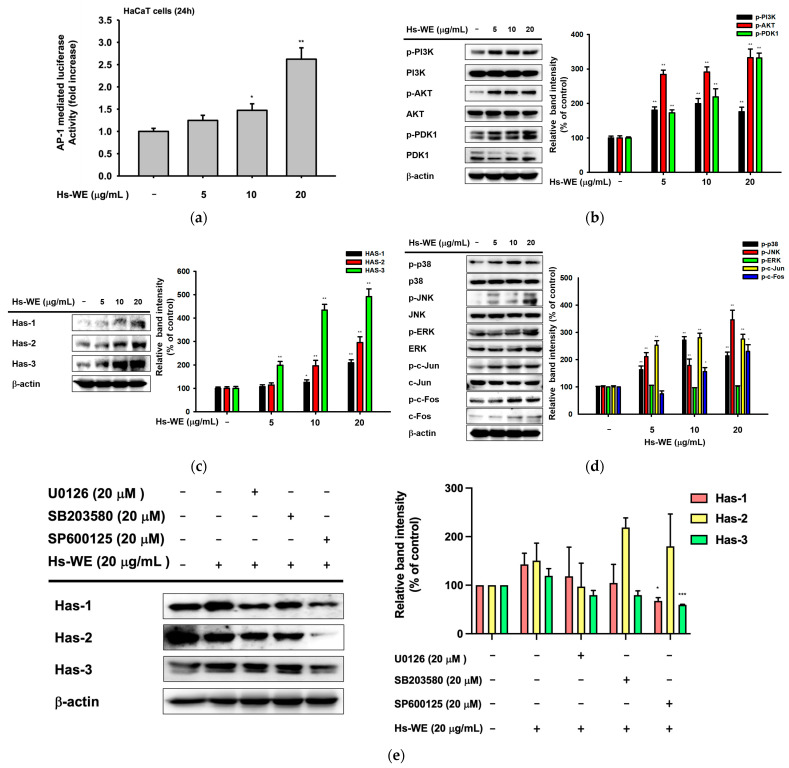
Effect of Hs-WE on the activation of AP-1 pathway. (**a**) Luciferase activity was determined with luminometer with lysates of HaCaT cells transfected with the AP-1-Luc and β-gal plasmids under the treatment of Hs-WE (5 to 20 µg/mL). (**b**–**e**) Levels of the Akt/PI3K pathway proteins (**b**), HAS proteins (**c**,**e**), and MAPK pathway proteins (**d**) were detected by Western blotting analysis with lysates of HaCaT cells incubated with Hs-WE (**b**–**d**) or MAPK inhibitors (U0126, SB203580, and SP600125) (**e**) for 24 h. (Values represent the mean ± standard deviation (SD) of three independent experiments. * *p* < 0.05, ** *p* < 0.01, and *** *p* < 0.001 compared with the normal group using Mann–Whitney U test.

A luciferase reporter assay was conducted to confirm the expression of AP-1 pathway proteins after Hs-WE treatment. At the transcription level, the AP-1-mediated luciferase expression was increased by Hs-WE treatment in a dose-dependent manner (Figure 4a). We also confirmed the change in expression of PI3K/AKT pathway proteins, which affect the NF-κB pathway. These results showed an increased pattern of phosphorylation for PI3K, AKT, and PDK-1 (Figure 4b). According to the clinical trial, we examined the protein levels of Has-1, 2, and 3 to determine if Has could be upregulated by treatment with Hs-WE at the protein levels. As we expected, the expressions of hyaluronic acid synthesis genes including Has-1, 2, and 3 were also increased at the protein level (Figure 4c). Previous reports have shown that enhanced AP-1 protein expression is associated with Has expression [19]. The increased expression of the proteins involved in the AP-1 signaling pathway was also shown through a Western blotting assay. We confirmed that the activated form of c-Jun and c-Fos increased at the protein level, as shown in the results of the luciferase reporter assay, which showed an increase in AP-1 levels. Next, we checked the upstream molecules of c-Jun and c-Fos. Their phosphorylation forms were upregulated in the case of JNK, ERK, and p38 (Figure 4d). Lastly, we examined the Has family protein levels after inhibitor and Hs-WE co-treatment. Has-1 and 3 levels were decreased even if co-treated with the Hs-WE and JNK inhibitors (Figure 4e). In summary, the skin moisturizing effect of Hs-WE is due to synergizing the upregulation of the AP-1 pathway and Akt/PI3K pathway proteins.

### 3.5. Hydrangenol Shows Skin Improvement Effects at the mRNA Level

**Figure 5:**
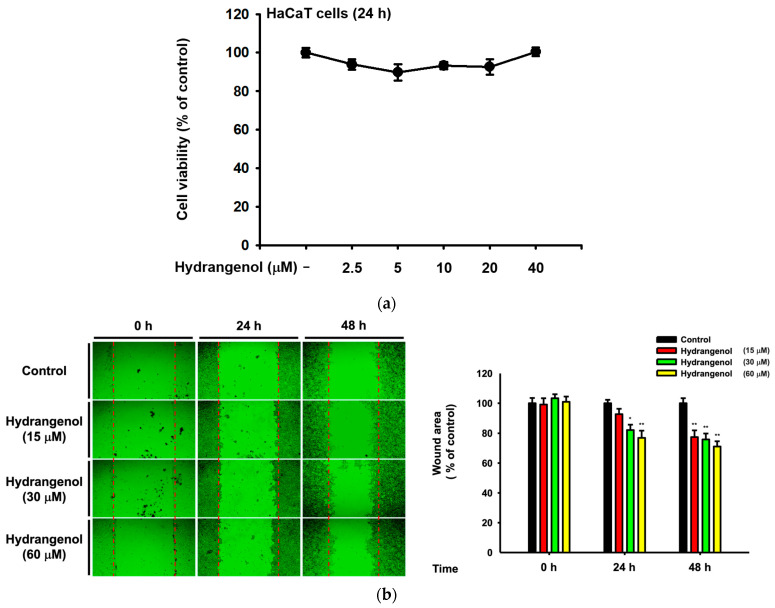

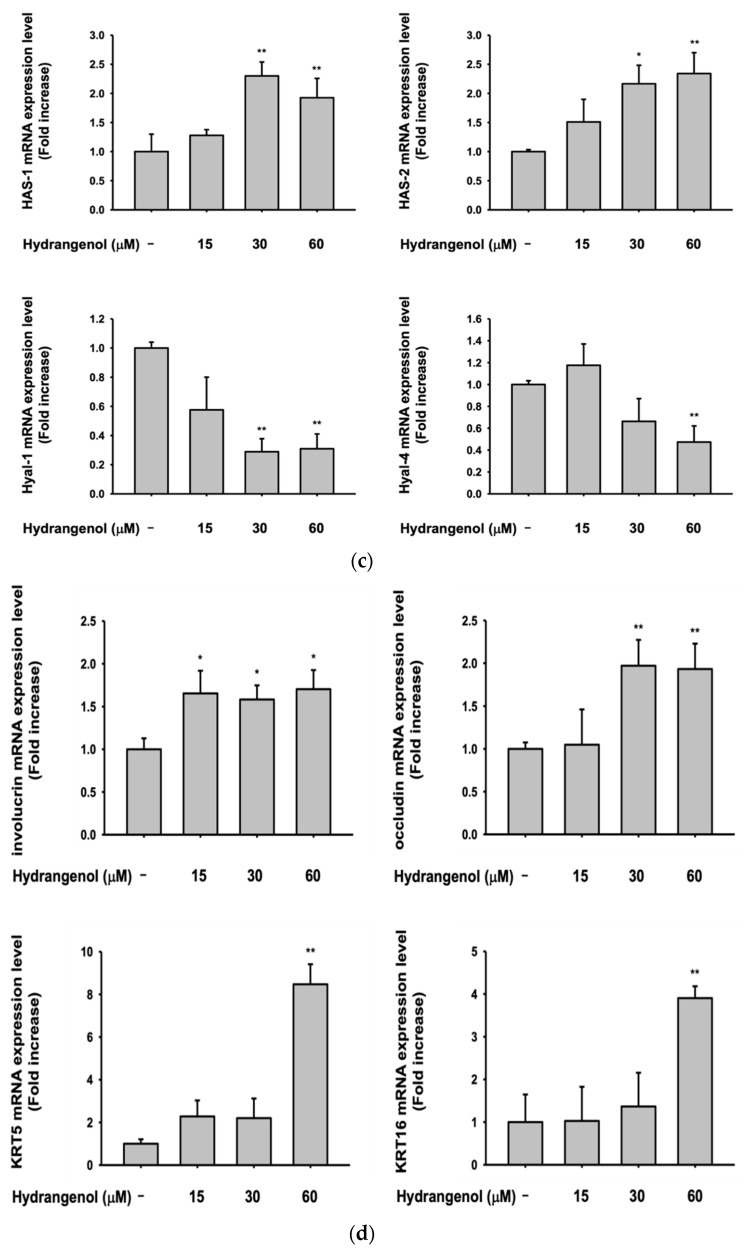
Effect of hydrangenol on the viability, wound healing, and expression of moisturizing factors and tight junction factors in HaCaT cells. (**a**) Cell viability of HaCaT cells in response to hydrangenol. The cells were plated in a 96-well plate. The cells were treated with hydrangenol for 24 h. (**b**) The wound-healing effects of hydrangenol were detected by comparing the normal and hydrangenol-treated groups. The pictures were taken at 0, 24, and 48 h. (**c**,**d**) The mRNA expressions were measured by real-time PCR. HaCaT cells were treated with hydrangenol for 24 h, and cDNA was synthesized after mRNA isolation. The mRNA levels of hyaluronic acid synthase and hyaluronidase (**c**), skin junction and skin proliferation factors (**d**) were detected. Values represent the mean ± standard deviation (SD) of three independent experiments. * *p* < 0.05, ** *p* < 0.01, compared with Hs-WE-treated group using the Mann–Whitney U test.

Hydrangenol a natural compound that can be isolated from hydrangeas. Hydrangenol is a representative natural compound of *H. serrata* used in many previous studies. Therefore, we investigated whether this compound also affects skin moisturization and cell growth to attenuate skin wrinkles. We conducted a conventional MTT assay to examine cell viability. The HaCaT cells showed no toxicity for all tested concentrations of hydrangenol (Figure 5a). Moreover, the wound area closed over 72 h in a dose-dependent manner (Figure 5b). We conducted a qRT-PCR assay to confirm these results with the hydrangenol treatment and found the same effect as the Hs-WE. We also examined the level of skin moisturizing factors and skin barrier-improving factors like the previous experiments. The transcription levels of these genes were improved by the presence of hydrangenol in a dose-dependent pattern (Figure 5c). The levels of keratin 5, 6, and 16 were increased by the treatment with hydrangenol (Figure 5d). This verifies the effects of the wound healing migration assay after hydrangenol treatment. Consequently, both hydrangenol and the Hs-WE showed significant effects, confirming that the effect of the Hs-WE was caused by hydrangenol.

### 3.6. Hydrangenol Improves Skin as the Main Component of Hs-WE

**Figure 6:**
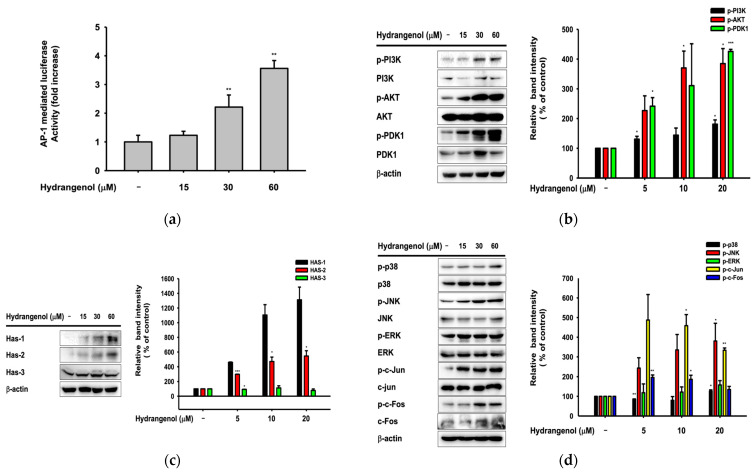

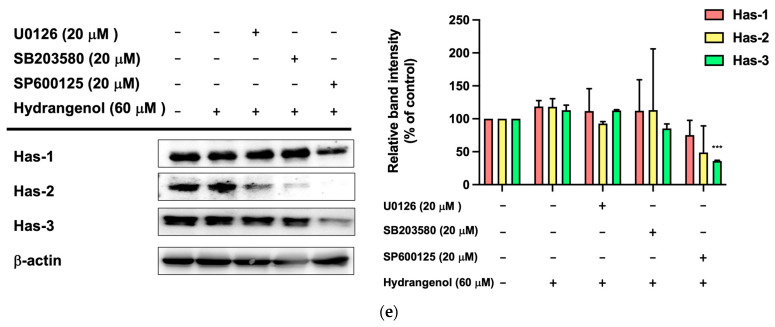
Effect of hydrangenol and MAPK inhibitors on the activation of AP-1 pathway and protein levels of moisturizing factors. (**a**) Luciferase activity in hydrangenol-treated HaCaT cells was determined with lumonometer by transfection with the AP-1-Luc and β-gal plasmids under treatment of hydrangenol (5, 10, and 20 µM). (**b**–**e**) Levels of PI3K/Akt pathway proteins (**b**), Has proteins (**c**,**e**), and MAPK pathway (**d**) were detected by Western blotting analysis with lysates of HaCaT cells treated with hydrangenol (**b**–**e**) or MAPK inhibitors (U0126, SB203580, and SP600125) (**e**) for 24 h. The relative intensity was measured by the ratio of phosphorylated protein expression/total expression of protein using ImageJ. Values represent the mean ± standard deviation (SD) of three independent experiments. * *p* < 0.05, ** *p* < 0.01, and *** *p* < 0.001 compared with the normal group using the Mann–Whitney U test.

We conducted a luciferase reporter assay to examine the Ap-1 transcription levels in hydrangenol-treated conditions. The Ap-1 mediated luciferase transcription level was upregulated in a dose-dependent pattern in transfected HaCaT cells (Figure 6a). We also investigated protein expression levels by performing Western blotting. Various studies have reported that the AKT/PI3K pathway promotes cell survival and transcription and blocks apoptosis by inactivating pro-apoptotic proteins [44,45]. In our results, phosphorylated AKT, PI3K, and PDK-1 was actively expressed in the hydrangenol-treated conditions (Figure 6b). This could be the mechanism for the wound healing activity of hydrangenol. Moreover, we checked the protein levels of HAS family genes in hydrangenol-treated cells. The expression patterns were increased in a dose-dependent patterns, suggesting that the AP-1 pathway could be involved in the upregulation of hyaluronic acid synthesis (Figure 6c). From these results, we could expect effects on the AP-1 protein signaling pathway. Meanwhile, c-Fos and c-Jun were significantly activated by hydrangenol, and the upstream proteins p38, ERK, and JNK were also upregulated in hydrangenol-treated cells (Figure 6d). Finally, we examined the HAS protein levels after treatment with JNK, p38, and ERK inhibitors. As we expected, HAS family protein expression levels decreased when JNK protein was inhibited by SP600125 (Figure 6e). Taken together, the activity of hydrangenol, which can upregulate AP-1 protein levels, especially through JNK, could assist the synthesis of HAS proteins.

## 4. Discussion

Keratinocytes are located at the outer-most layer of the skin and play an important role in protecting the skin from external stimuli, including UV irradiation, reactive oxidative species, fine dust, and air pollution. Skin aging can be accelerated by these stimuli, and skin aging caused by these factors is referred to as extrinsic aging [46,47]. Aging that occurs naturally over time is called natural aging or endogenous aging. Accordingly, the symptoms of aging also appear different depending on which factors are involved. In the case of intrinsic aging, symptoms like reduction of the skin dermis, relief of flexions between the epidermis and dermis, increased moisture evaporation in the skin, and fine wrinkle formation were observed [1]. Skin hydration is essential to retain skin homeostasis [48]. Skin dryness can cause skin wrinkles, and decreased skin moisture also affects skin cells. Many studies about anti-aging focused on two mechanisms. One is the upregulation of skin moisturizing factors. They improved hyaluronic acid synthesis (such as upregulating the HAS family proteins) to maintain good skin conditions or prevent skin dryness by inhibiting hyaluronidases. The other is the reinforcement of the skin barrier component. By upregulating the skin junction factors like FLG, TGM-1, OCLN, INVN, and the keratin family, we can reduce transepidermal water loss from the skin. Various trials have been conducted to upregulate the Has genes and skin barrier factors, and downregulate hyaluronidase to prevent symptoms. Previous reports have investigated the relationship between the AP-1 pathway and skin barrier-restoring mechanisms [18]. *H. serrata* is a plant affiliated with hydrangeaceae. It is called “san soo gook” because it is a plant native to mountainous areas including Korea and Japan. It has been used for tea because of its sweet taste. Previous studies demonstrated the anti-obesity effects, muscle synthesis effects, and anti-photoaging effects of *H. serrata* [39,40,41,42,43]. We investigated the skin improvement potential of a Hs-WE. Skin moisturizing effects as well as anti-wrinkle effects were observed in the 0.5% Hs-WE-treated group (Figure 1a–c). In a quantitative analysis experiment, LC/MS was conducted to identify the diverse compounds in the Hs-WE. The results of this experiment revealed the main component of Hs-WE and other chemicals such as coumarin compounds, polyphenols, and flavonoids (Figure 2b). Previous studies showed that various metabolites in plants help in improving skin health [49,50], In the case of melatonin, for example, not only is it a substance found in natural honey [51], it is also known to have a skin-protective role by reducing transepidermal water loss (TEWL) [52]. Moreover, vitamin D3, which is found in natural honey, has also been reported as a natural anti-aging component [53,54]. We assumed that the results of clinical trials may also be the result of various phytochemicals in the Hs-WE. HaCaT cells were used in all experiments to identify more specific mechanisms of Hs-WE in skin cells. First, the Hs-WE showed no toxicity to cells even at a high concentration of 200 µg/mL (Figure 2a). The wound healing migration assay results confirmed that the Hs-WE could potentially prevent wrinkles by inducing cell proliferation without toxicity in epidermal cells (Figure 2d). Skin dryness can be prevented by decreasing the water loss from weakened skin barriers. Factors such as TGM, OCLN, INVN, and FLG reinforce the skin junction and skin barrier [55,56,57]. The presence of the Hs-WE upregulated both signal transduction to synthesize moisturizing factors such as HAS1, 2, and 3 (Figure 3a) and skin barrier factors such as TGM, occludin, involucrin, and filaggrin (Figure 3c). The keratin 5, 6, and 16 levels were increased by treatment with Hs-WE (Figure 3). These three genes contribute to cell proliferation and skin turnover. COL1A1 was also upregulated at the both mRNA and protein levels when HDF cells were treated with Hs-WE or hydrangenol (Figure 3e,f). From these results, we hypothesized the wound-healing effect of the Hs-WE was due to the increased keratin 5, 6, and 16. Additionally, we also hypothesized that the Hs-WE can increase collagen synthesis in the dermis. AP-1 plays a pivotal function in the epidermis, repairing wounds by differentiation. Meanwhile, the upstream molecule p38 MAPK induces Has1 transcription. Protein levels of PI3k, Akt, MAPK, and AP-1 were confirmed in the presence of the Hs-WE (Figure 4b,d) or hydrangenol (Figure 6b,d). When the inhibitors of MAPK were added to HaCaT cells, the key molecules which induce *HAS* expression were increased after the treatment with Hs-WE and hydrangenol (Figure 4e and Figure 6e). Considering the previous results in which various phytochemicals were detected in the Hs-WE, we hypothesized that the synergistic effect is due to the biochemicals in the extract. We confirmed that hydrangenol, an indicator component of Hs-WE, also acts on the skin in the same way.

Our study had some limitations including the fact that primary human keratinocytes were not used in the experiments. Many papers dealing with skin research have been published about the differences in gene expressions and protein levels between NHEK and HaCaT cells [58,59,60]. Since the expression levels may be increased or decreased when the same experiments are conducted with normal human keratinocytes, the results observed in both cell types is necessary. Moreover, histological approaches with human-derived skin tissues biopsied from clinical trials or three-dimensional culture conditions will be also necessary to confirm the activity of the Hs-WE on the reduction of wrinkles and its mechanisms. Despite these technical limitations, however, we believe that the Hs-WE is effective in the improvement of wrinkle formation according to the clinical trial and in the upregulation of moisturizing factors by measuring the relevant proteins at the mRNA and protein levels, suggesting that it can be applied as a cosmeceutical and nutritional supplement for human skin. Nonetheless, to support our current data, we will further verify the Hs-WE’s effect using fresh primary dermal keratinocytes and human skin. Therefore, these results seem to prove that the steady and repeated application of hydrangea extract produces anti-wrinkle effects by upregulating moisturizing factors and skin barrier constituents without skin toxicity.

## 5. Conclusions

**Figure 7:**
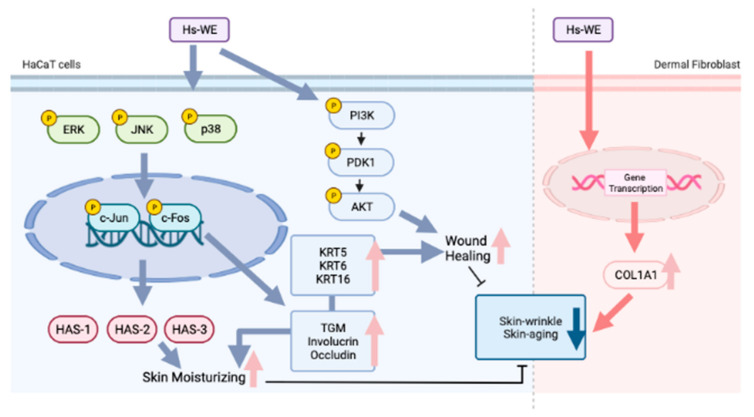
Summary of Hs-WE’s effect on keratinocytes and fibroblasts. This figure was created with BioRender.com.

In our study, skin cell viability and proliferation were enhanced by treatment with the Hs-WE or hydrangenol. The moisturizing effects of the Hs-WE or hydrangenol were confirmed by detecting factors such as Has and Hyal at the mRNA level. The attenuating effects on skin wrinkles were also examined by measuring the mRNA levels of skin junction, procollagen, and proliferation factors. Proteins, including AP-1, Akt, and Has, were upregulated by treatment with the Hs-WE or hydrangenol. Additionally, Has-1, 2, and 3 expression levels were commonly inhibited in the presence of JNK inhibitors. Taken together, the Hs-WE and hydrangenol could enhance these protein levels via the phosphorylation of JNK. We concluded that the Hs-WE and hydrangenol can increase skin moisture levels and attenuate skin wrinkles by promoting the proliferation of keratinocytes. Therefore, we suggested that the Hs-WE could improve skin wrinkles and dryness via routine application to the skin (Figure 7). For this purpose, we are planning to develop a cosmetic preparation with 0.5% or more of Hs-WE or its food formulation with skin health functionality to be used or taken twice a day, usually in the morning and evening. Finally, since providing sufficient nutrients to the skin is important to prevent wrinkles and skin hydration, the development of cosmetic formulations is an important issue to enhance the penetration of nutrients. Therefore, we will continuously study to develop effective cosmeceutical preparations or even functional foods.

## Figures and Tables

**Table 1:** The sequences of primers used in qRT-PCR.

Gene Name		Sequence (5′–3′)
*HAS-1*	Forward	GTATCCTGCATCAGCGGTCC
	Reverse	TGCCGGTCATCCCCAAAAG
*HAS-2*	Forward	CCTCCTGGGTGGTGTGATTT
	Reverse	GCGTCAAAAGCATGACCCAA
*HAS-3*	Forward	CAAGTGCCTCACAGAGACCC
	Reverse	GGAAGAAACCCGTGACCACT
*HYAL-1*	Forward	CAGAATGCAGCCTGATTGC
	Reverse	CCGGTGTAGTTGGGGCTTAG
*HYAL-2*	Forward	TACACCACAAGCACGGAGAC
	Reverse	ATGCAGGAAGGGTACTGGCAC
*HYAL-3*	Forward	CCAGGATGACCTTGTGCAGT
	Reverse	CCATCTGTCCTGGATCTCGC
*HYAL-4*	Forward	TGACCTCTCTTGGCTCTGGA
	Reverse	AGGCAGCACTTTCTCCTATGG
*Ker5*	Forward	GAGATCAGTGACTTGTGCGT
	Reverse	ATTGCTGAGTTGCTCAGGTG
*Ker6*	Forward	TCCTCTTCGAGCCGTCAGA
	Reverse	TGGTAGAGGCAGCTCAGTTC
*Ker16*	Forward	CCAGGGACTGATTGGCAGTGT
	Reverse	AAGGGTCTGGGAGGCAGAACT
*TGM-1*	Forward	ACAGGCTCATCTGGTTGGTG
	Reverse	TTCCCGATGCTTGTGGTCTC
*FLG*	Forward	TGAGGCATACCCAGAGGACT
	Reverse	CTGTATCGCGGTGAGAGGAT
*OCLN*	Forward	TGGCCTACAGGAATACAAGAGC
	Reverse	AAAGGATGCTGTACCTCCACAG
*IVLN*	Forward	CCAGAAGGTGCCTGTCGAG
	Reverse	TCAGGCAGTCCCTTTACAGC
*COL1A1*	Forward	CAGGTACCATGACCGAGACG
	Reverse	AGCACCATCATTTCCACGAG

**Table 2:** Inclusion criteria and exclusion criteria used for the validity of clinical trials.

Criteria	No.	Contents
Inclusion criteria	1	Females with dried skin, aged 30–59 years (average = 49.2 years).
	2	The subject has eye wrinkles (crow’s feet).
	3	A person who has voluntarily signed consent after the test purpose and content were fully explained.
	4	Those who can follow up during the test period.
	5	A healthy person without an acute or chronic physical disease including skin diseases.
Exclusion criteria	1	Pregnant or lactating women and women of childbearing age who do not agree to the contraceptive method prescribed by the protocol.
	2	A person with a lesion at the test site or suffering from an infectious skin disease.
	3	People with allergies, hypersensitivity, or irritation to cosmetics, pharmaceuticals, or daily exposure to light.
	4	Those who have received systemic steroids or phototherapy within 1 month of participating in the trial, or who have received skin treatments (scaling/Botox/filler/laser/tattoo) within 3 months of participating in the trial.
	5	Those who have used drugs with similar functions at the research site within 3 months before the start of the study, or have a mental illness or mental retardation disorder.
	6	Other than the above, a person who will make it difficult to conduct a human test based on the judgment of the responsible researcher or the person in charge of the study.

**Table 3:** The ingredients used in making clinical trial samples for placebo samples.

No.	Ingredient Name
1	Water
2	Glycerin
3	Butylene glycol
4	Dipropylene glycol
5	Disodium EDTA
6	Triethanolamine
7	Cetearyl alcohol
8	Caprylic/capric triglyceride
9	Glyceryl stearate
10	PEG-100 stearate
11	Carbomer
12	1,2-hexanediol
13	Phenoxyethanol
14	Sodium acrylate/sodium acryloyldimethyl taurate copolymer
15	Polyisobutene
16	Sorbitan oleate
17	Caprylyl/capryl glucoside

**Table 4:** The ingredients used in making clinical trial samples for Hs-WE 0.5% samples.

No.	Ingredient Name
1	Water
2	Glycerin
3	Butylene glycol
4	Dipropylene glycol
5	Disodium EDTA
6	Triethanolamine
7	Cetearyl alcohol
8	Caprylic/capric triglyceride
9	Glyceryl stearate
10	PEG-100 stearate
11	Carbomer
12	1,2-hexanediol
13	Phenoxyethanol
14	Sodium acrylate/sodium acryloyldimethyl taurate copolymer
15	Polyisobutene
16	Sorbitan oleate
17	Caprylyl/capryl glucoside
18	*Hydrangea serrata* leaf extract

**Table 5:** The measured results of skin wrinkle measurement in the clinical study.

		0.5% Hs-WE	Placebo
Moisturizing measurement value (A.U)	0 week	51.544 ± 9.862	51.771 ± 10.593
	after 2 weeks	54.828 ± 10.214	53.230 ± 10.631
	after 4 weeks	58.525 ± 10.735	55.705 ± 10.147
Variance ^a^	0 week–after 2 weeks	3.284	1.459
	0 week–after 4 weeks	6.981	3.934
Improvement rate ^b^ (%)	0 week–after 2 weeks	6.371	2.818
	0 week–after 4 weeks	13.544	7.599
*p*-Value within group	0 week–after 2 weeks	<0.001	<0.001
	0 week–after 4 weeks	<0.001	<0.001
*p*-Value between groups	0 week–after 2 weeks	<0.001	
	0 week–after 4 weeks	<0.001	

^a^ Variance: (value after using the cream—value before using the cream). ^b^ Improvement rate: $\left|\left\{(\mathrm{value}\;\mathrm{after}\;\mathrm{using}\;\mathrm{the}\;\mathrm{cream}-\mathrm{value}\;\mathrm{before}\;\mathrm{using}\;\mathrm{the}\;\mathrm{cream})/\mathrm{value}\;\mathrm{before}\;\mathrm{using}\;\mathrm{the}\;\mathrm{cream}\times 100\right\}\right|.$

**Table 6:** The measured results of skin moisture in the clinical study.

		0.5% Hs-WE	Placebo
Measurement of skin wrinkles (depth, mm)	0 week	0.102 ± 0.029	0.094 ± 0.030
	after 2 weeks	0.087 ± 0.027	0.087 ± 0.030
	after 4 weeks	0.074 ± 0.023	0.081 ± 0.029
Variance ^a^	0 week–after 2 weeks	−0.015	−0.007
	0 week–after 4 weeks	−0.028	−0.013
Improvement rate ^b^ (%)	0 week–after 2 weeks	14.706	7.447
	0 week–after 4 weeks	27.451	13.830
*p*-value within group	0 week–after 2 weeks	<0.001	<0.001
	0 week–after 4 weeks	<0.001	<0.001
*p*-value between groups	0 week–after 2 weeks	0.002	
	0 week–after 4 weeks	<0.001	

^a^ Variance: (value after using the cream—value before using the cream). ^b^ Improvement rate: $\left|\left\{(\mathrm{value}\;\mathrm{after}\;\mathrm{using}\;\mathrm{the}\;\mathrm{cream}-\mathrm{value}\;\mathrm{before}\;\mathrm{using}\;\mathrm{the}\;\mathrm{cream})/\mathrm{value}\;\mathrm{before}\;\mathrm{using}\;\mathrm{the}\;\mathrm{cream}\times 100\right\}\right|.$

**Table 7:** The natural compounds in Hs-WE verified through LC/MS-MS analysis.

Peak No.	RT	Name of the Compound
1	1	GABA
2	1.94	Gallic acid
3	3.85	Protocatechuic acid
4	6.87	Chlorogenic acid
5	7.59	Vanillic acid
6	7.8	Caffeic acid
7	8.61	Loganin_A
8	9.68	p-Coumaric acid
9	10.42	Rutin
10	10.56	Ferulic acid
11	10.74	Hyperoside
12	10.89	Isoquercitrin
13	11.11	Salicylic acid
14	11.8	Astragalin
15	13.42	Resveratrol
16	14.78	Hydrangenol
17	16.75	Kaempferol

## Data Availability

Data will be made available on request.

## Abbreviation

ROS	reactive oxygen species
HDF	human dermal fibroblast
HYAL	hyaluronidase
HAS-1	hyaluronic acid synthase 1
HAS-2	hyaluronic acid synthase 2
HAS-3	hyaluronic acid synthase 3
INVN	involucrin
OCLN	occludin
TGM	transglutaminase 1
FLG	filaggrin
AP-1	activator protein 1
MAPK	mitogen-activated protein kinase
ERK	extracellular signal-regulated kinases
JNK	c-Jun NH2-terminal kinases
HaCaT cells	human keratinocyte cell line
DMEM	Dulbecco’s modified eagle’s medium
PC/SM	penicillin/streptomycin solution
FBS	fetal bovine serum
MTT	3-(4,5-dimethylthiazol-2-yl)-2,5-diphenyltetrazolium bromide
